# Supplementary material for: Induction of open-form bile canaliculus formation by hepatocytes for evaluation of biliary drug excretion
Source: Commun Biol. 2023 Aug 22;6:866. doi: 10.1038/s42003-023-05216-z (PMC10444810; doi:10.1038/s42003-023-05216-z)
Supplement: Supplementary file 2 — Supplementary Information [file 42003_2023_5216_MOESM2_ESM.pdf]

Supplementary Information

Induction of open-form bile canaliculus formation by hepatocytes for evaluation of biliary drug excretion

Hiroshi Arakawa[1,‡], Yuya Nakazono[1,‡], Natsumi Matsuoka[1], Momoka Hayashi[1], Yoshiyuki Shirasaka[1], Atsushi Hirao[2,3], and Ikumi Tamai[1,*]

[1]Faculty of Pharmaceutical Sciences, Institute of Medical, Pharmaceutical and Health Sciences, Kanazawa University, Kanazawa 920-1192, Japan

[2]Division of Molecular Genetics, Cancer Research Institute, Kanazawa University, Kakuma-machi, Kanazawa 920-1192, Japan.

[3]WPI Nano Life Science Institute (WPI-Nano LSI), Kanazawa University, Kakuma-machi, Kanazawa 920-1192, Japan

[‡]These authors contributed equally to this work.

* To whom corresponding to

Department of Membrane Transport and Biopharmaceutics, Faculty of Pharmaceutical Sciences, Kakuma-machi, Kanazawa 920-1192, Japan.

Tel: +81-76-234-4479, Fax: +81-76-264-6284,

E-mail: tamai@p.kanazawa-u.ac.jp

Supplementary Results

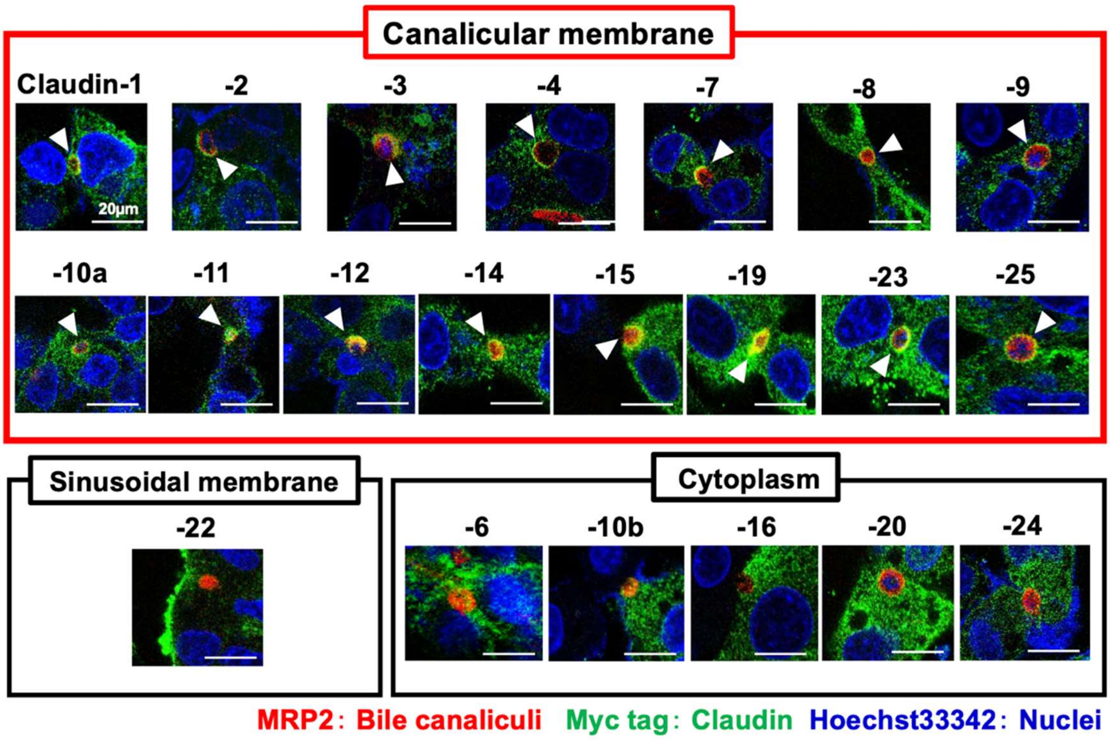

Supplementary Fig.1: Localization of claudin families in HepG2 cells. Immunofluorescent staining of the bile canaliculi marker MRP2 (red) was observed with co-staining of the myc tag of each claudin (green) and the nuclei marker hoechst33342 (blue). White arrowhead indicates co-staining of the bile canaliculi marker MRP2 and the claudin marker myc tag. Bar = 20 μm.

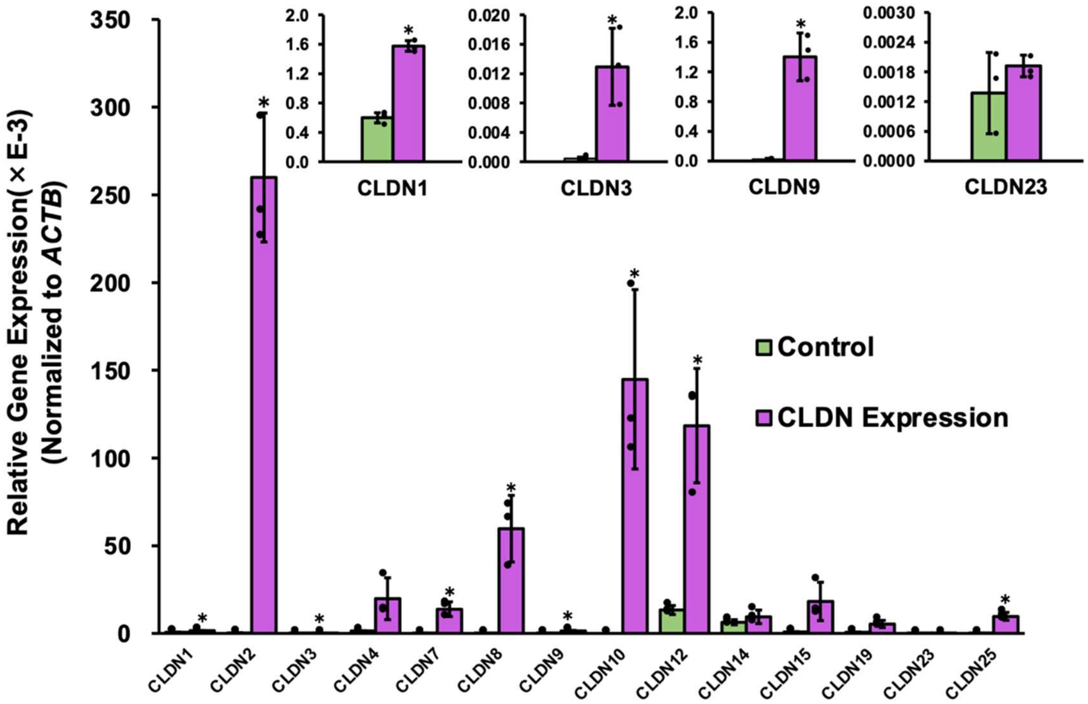

Supplementary Fig.2: Claudin gene transduction using lentiviral method in HepG2 cells. The green and purple bars show the expression levels of claudins in the Mock and CLDN gene transfected HepG2 cells, respectively. The bars represent the means of the corresponding group, and the error bars represent the S.D. (n = 3 biological replicate wells). Claudin gene expression was normalized to *ACTB*.

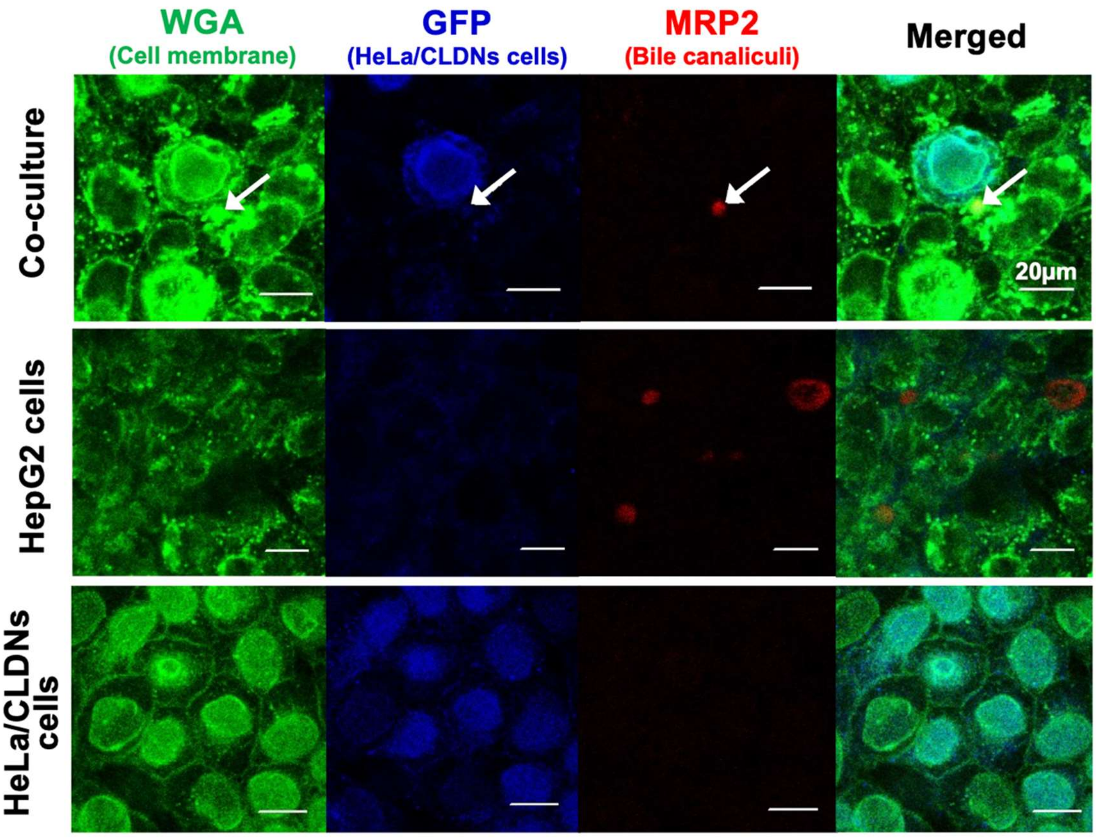

Supplementary Fig.3: Immunocytochemical detection of formation of bile canaliculi in the co-culture of HeLa/CLDN-1, -2, -3, -9 and HepG2/CLDN-1, -2, -3, -9. Immunofluorescent staining of MRP2 (red) with co-staining of GFP (blue) as HeLa cell marker and WGA (green) as plasma membrane marker in each cell was performed. White arrowhead shows MRP2 staining localized between GFP expressing cells and non-GFP expressing cells. Bar = 20 μm.

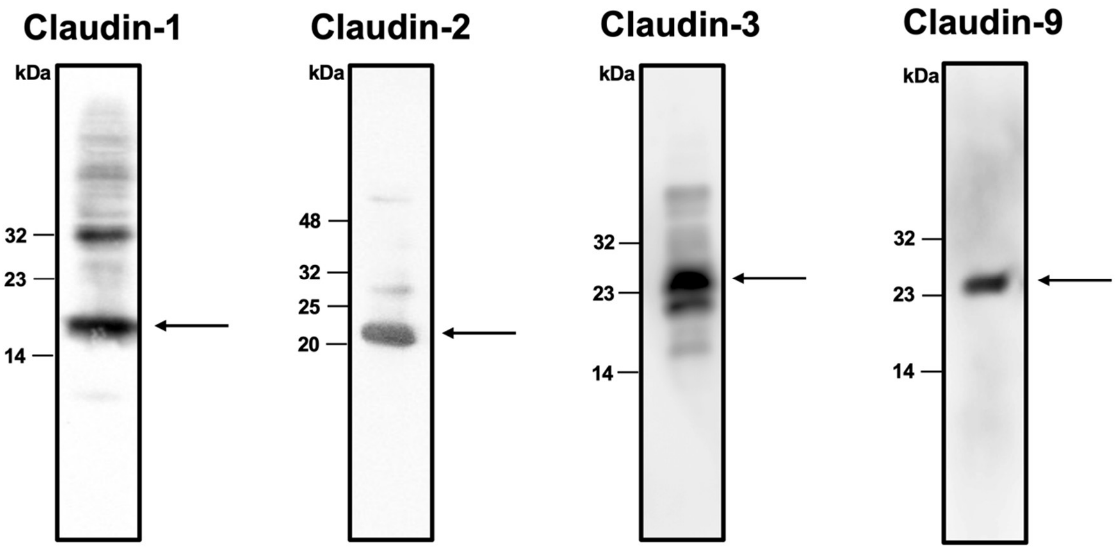

Supplementary Fig.4: *In vitro* protein synthesis of claudin-1, -2, -3, and -9. The protein of claudin-1, -2, -3, and -9 was synthesized by cell-free S-MF method and detected by western blotting. The black arrows represent bands of each claudin protein estimated by molecular weight.

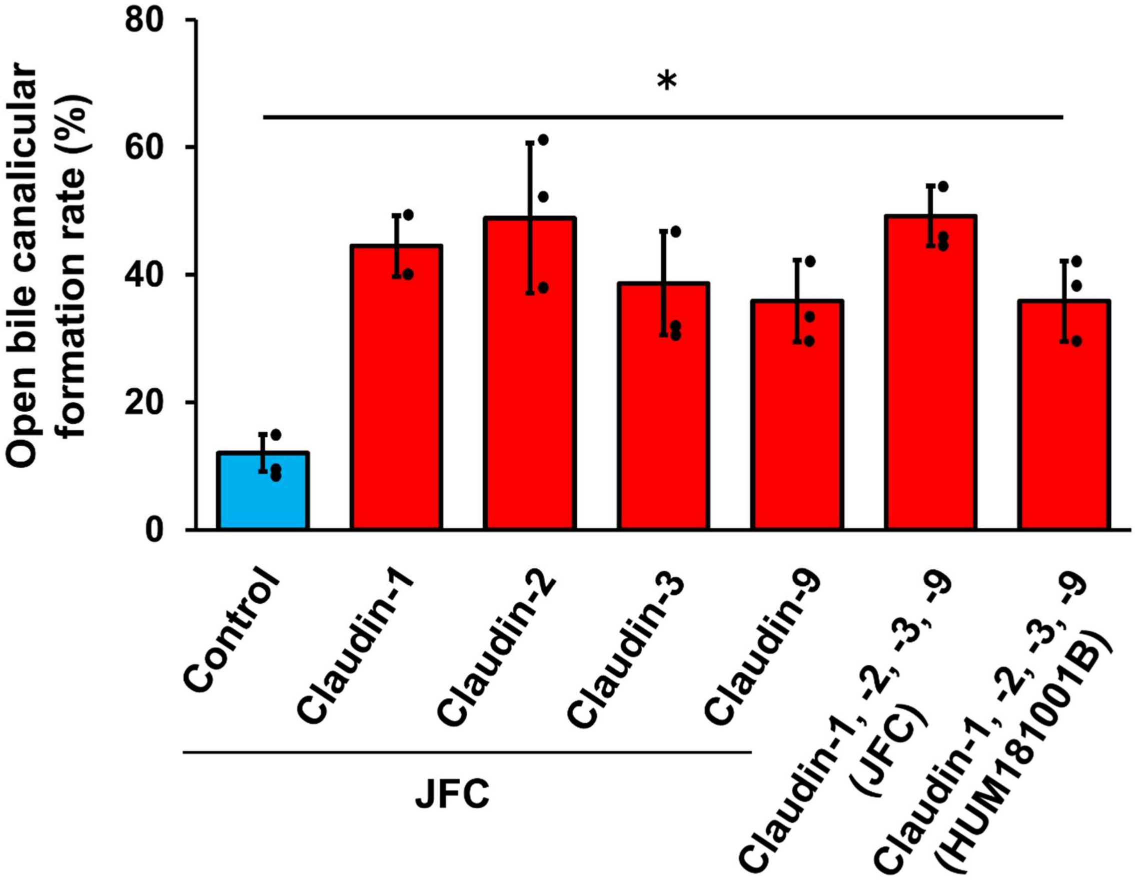

Supplementary Fig.5: Rate of open bile canaliculus formation in PXB-cells cultured on claudin-coated plates. Number of localized bile canaliculi lumen face to culture plate (%) in PXB-cells (JFC, HUM181001B) cultured on control (collagen-coated) (green) and claudin-coated (red) plates, respectively (n = 3 biological replicate wells). Data are represented as the means ± S.D. Statistical significance was determined using Student's *t*-test; *$P < 0.05$.

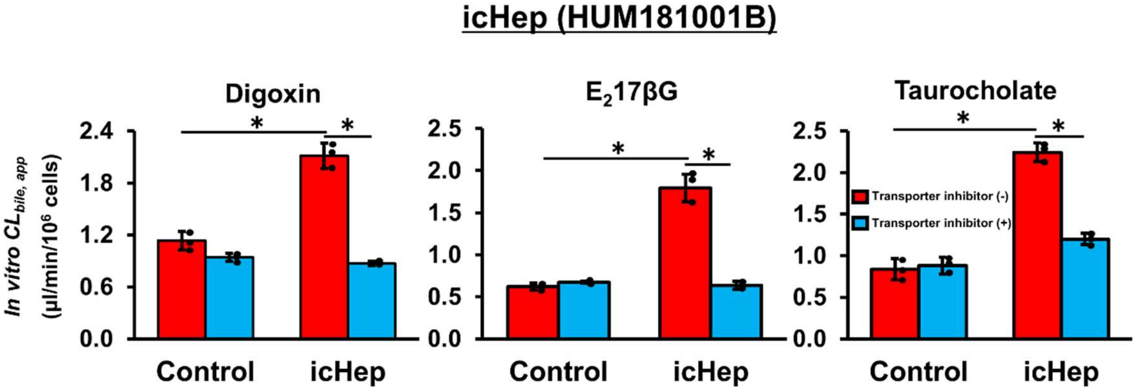

Supplementary Fig.6: Biliary transporter-mediated transport of each typically substrate in icHep (HUM181001B). icHep were cultured on a permeable support for 7 days. The biliary transport of digoxin, $E_2 17\beta G$, and taurocholate across the cells was measured from the sinusoidal side to the bile side. icHep were incubated with (left) $[^3H]$digoxin (1 µCi/ml), (center) $[^3H]E_2 17\beta G$ (1 µCi/ml), or (right) $[^3H]$taurocholate (1 µCi/ml) on the sinusoidal side for 120 min in the absence (red bars) or presence (green bars) of (left) the P-gp inhibitor zosuquidar (5 µM), (center) MRP2 inhibitor benzbromarone (100 µM), or (right) BSEP inhibitor chlorpromazine (30 µM), respectively. Data are presented as the means ± S.D. (n = 3 biological replicate wells). Statistical significance was determined using the Tukey-Kramer test; $*P < 0.05$.

a.

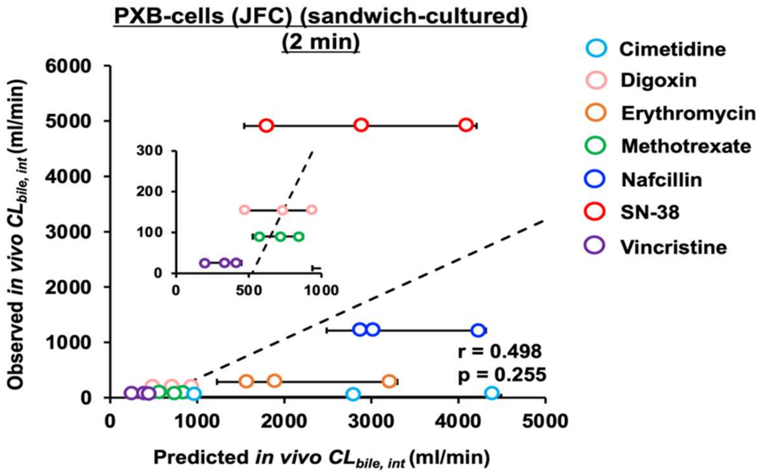
PXB-cells (JFC) (sandwich-cultured)
(2 min)
Observed in vivo CLbile, int (ml/min)
Predicted in vivo CLbile, int (ml/min)
0
1000
2000
3000
4000
5000
6000
0
1000
2000
3000
4000
5000
300
200
100
0
0
500
1000
r = 0.498
p = 0.255
Cimetidine
Digoxin
Erythromycin
Methotrexate
Nafcillin
SN-38
Vincristine

b.

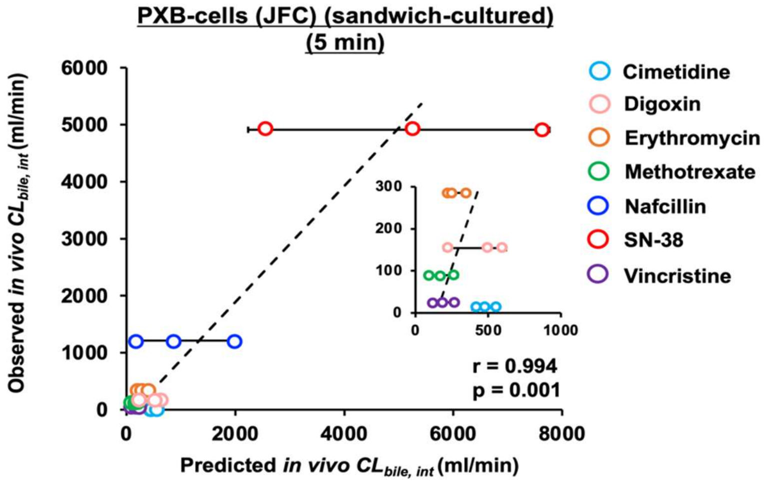
PXB-cells (JFC) (sandwich-cultured)
(5 min)
Observed in vivo CLbile, int (ml/min)
Predicted in vivo CLbile, int (ml/min)
0
1000
2000
3000
4000
5000
6000
0
2000
4000
6000
8000
300
200
100
0
0
500
1000
r = 0.994
p = 0.001
Cimetidine
Digoxin
Erythromycin
Methotrexate
Nafcillin
SN-38
Vincristine

c.

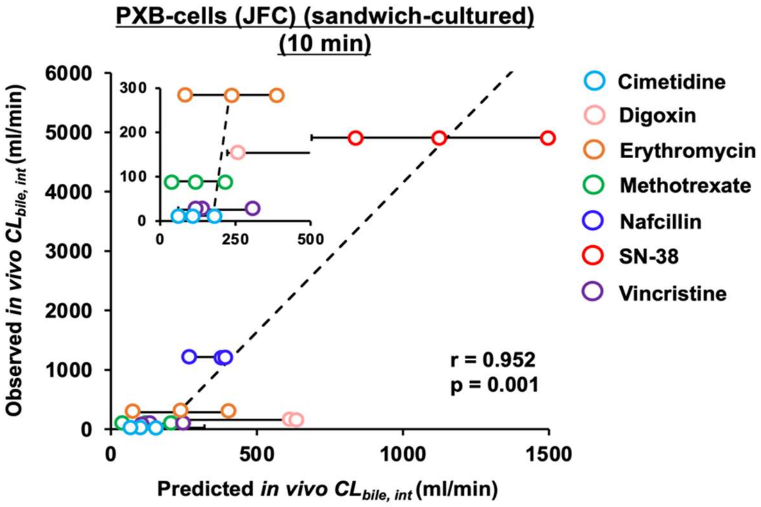
PXB-cells (JFC) (sandwich-cultured)
(10 min)
Observed in vivo CLbile, int (ml/min)
Predicted in vivo CLbile, int (ml/min)
0
1000
2000
3000
4000
5000
6000
0
500
1000
1500
300
200
100
0
0
250
500
r = 0.952
p = 0.001
Cimetidine
Digoxin
Erythromycin
Methotrexate
Nafcillin
SN-38
Vincristine

d.

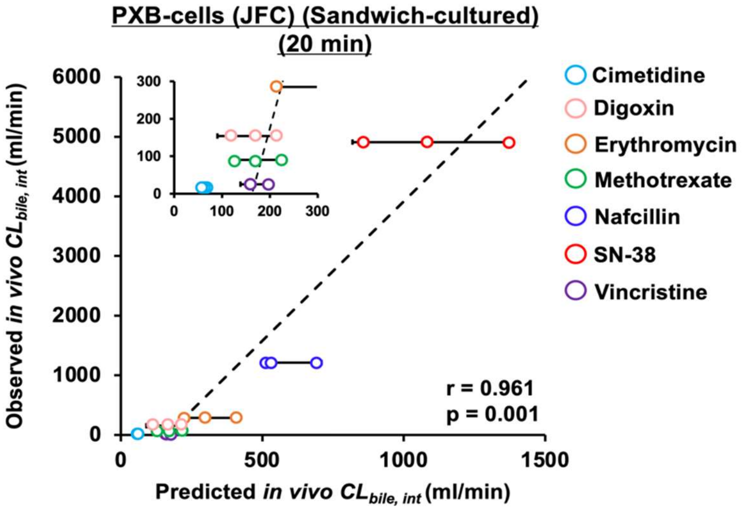

Supplementary Fig.7: Prediction of human *in vivo* biliary excretion clearance using sandwich-cultured PXB-cells. a, **b**, **c**, **d** The correlation between *in vitro* [sandwich-cultured PXB-cells (JFC)] and *in vivo* biliary clearance of test compounds (cimetidine, digoxin, erythromycin, methotrexate, nafcillin, SN-38, and vincristine) was evaluated. *In vivo* biliary clearance was estimated from the BEI (%) calculated for (a) 2, (b) 5, (c) 10, and (d) 20 min incubations of each test compound. Predicted *in vivo* data are presented as the means ± S.D. (n = 3 biological replicate wells). Statistical significance was determined by Pearson's correlation analysis.

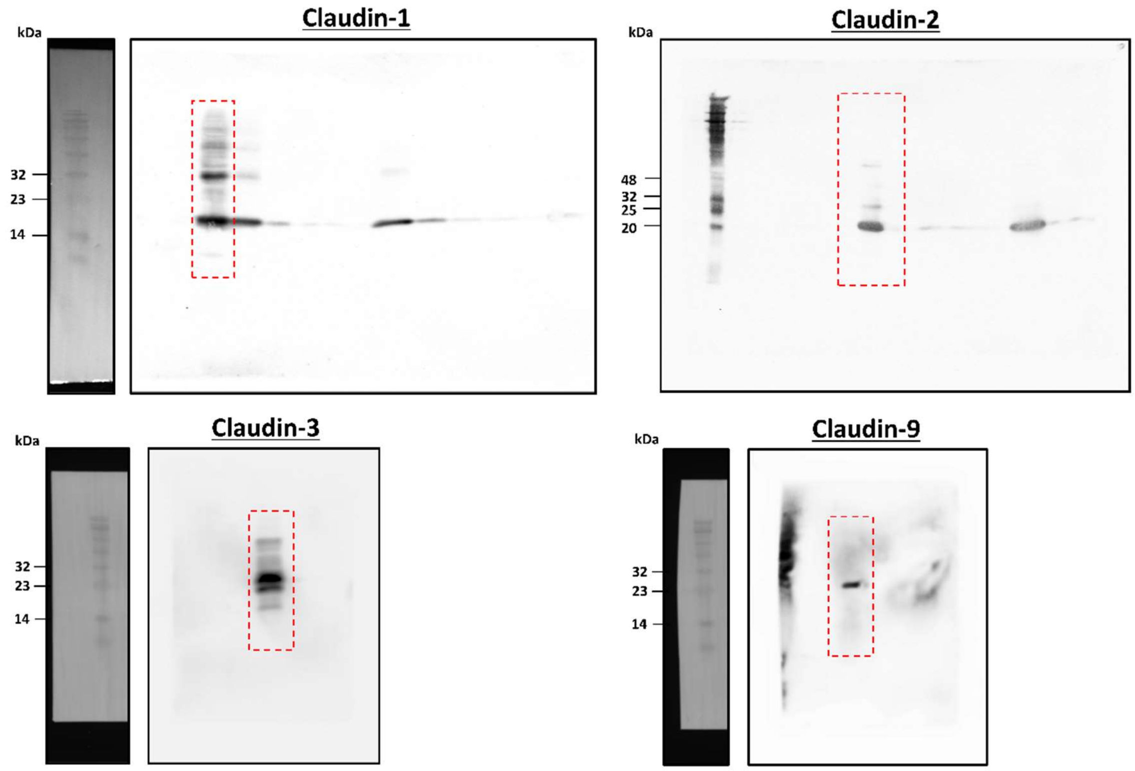

Supplementary Fig.8: Uncropped scans of western blots.

Unedited western blots of Supplementary Fig.4. Blots are displayed with molecular marker.

Supplementary Table 1: Component of the CYP substrate cocktail and its metabolites.

Enzymes	Probe substrates	Concentration in reaction mixture (μM)	Metabolites
CYP1A2	Phenacetin	20	Acetaminophen
CYP2B6	Bupropion	10	Hydroxybupropion
CYP2C9	Diclofenac	1	4'-hydroxydiclofenac
CYP2C19	(S)-mephenytoin	40	4'-hydroxymephenytoin
CYP2D6	Bufuralol	5	1'-hydroxybufuralol
CYP2E1	Chlorzoxazone	40	6'-hydroxychlorzoxazone
CYP3A4	Midazolam	5	1'-hydroxymidazolam
UGT1A1	SN-38	10	SN-38G

Supplementary Table 2: Human hepatocyte donor demographics.

Donor ID	Sex	Race	Age	Viability (%)
HU1663	Male	Caucasian	56	94
JFC	Male	Caucasian	1	94
HUM181001B	Female	Caucasian	12	96

Supplementary Table 3: Antibody list for immunofluorescence (IF) and western blot (WB) analysis.

Product Name	Cat No	Supplier	Assay Conc
anti-MRP2 antibody, mouse IgG monoclonal	GTX23373	Gene Tex (Irvine, CA, U.S.A.)	IF (1:100)
anti-claudin-1 antibody, mouse IgG monoclonal	sc-81796	Santa Cruz Biotechnology (Dallas, CA, U.S.A.)	IF (1:50), WB (1:200)
anti-claudin-2 antibody, rabbit IgG polyclonal	ab53032	abcam (Cambridge, U.K.)	WB (1:500)
anti-claudin-3 antibody, rabbit IgG monoclonal	ab214487	abcam	WB (1:1000)
anti-claudin-9 antibody, mouse IgG monoclonal	sc-398836	Santa Cruz Biotechnology	WB (1:100)
anti-P-gp antibody, rabbit IgG monoclonal	13342S	Cell Signaling Technology (Danvers, MA, U.S.A.)	IF (1:400)
anti-BSEP antibody, rabbit IgG polyclonal	HPA019035	Sigma-Aldrich	IF (1:500)
anti-OATP1B1 antibody, mouse IgM monoclonal	ab15441	abcam	IF (1:100)
anti-OATP1B3 antibody, rabbit IgG polyclonal	HPA004943	Sigma-Aldrich	IF (1:100)
anti-NTCP antibody, rabbit IgG polyclonal	ab131084	abcam	IF (1:100)
anti-OCT1 antibody, mouse IgG monoclonal	NBP1-51684	Novus Biologicals (Littleton, CO, U.S.A.)	IF (1:400)
Goat anti-mouse Alexa Fluor 594	A11005	Thermo Fisher Scientific	IF (1:200)
Goat anti-mouse IgG (H+L) secondary antibody HRP	62-6520	Thermo Fisher Scientific	WB (1:5000)
Goat anti-rabbit IgG (H+L) secondary antibody HRP	65-6120	Thermo Fisher Scientific	WB (1:3000)
Donkey anti-rabbit Alexa Fluor 488	A21206	Thermo Fisher Scientific	IF (1:1000)
Goat anti-mouse Alexa Fluor 488	A11001	Thermo Fisher Scientific	IF (1:1000)
Goat anti-mouse Alexa Fluor 488	ab150121	abcam	IF (1:1000)

Supplementary Table 4: Primer list.

Gene	Forward primer	Reverse primer
hCLDN1	5'-TGTCATTGGGGGTGCGATA-3'	5'-AGGAACAGCAAAGTAGGGCA-3'
hCLDN2	5'-AAAGACAGAGTGGCGGTAGC-3'	5'-CAGTGGTGAGTAGAAGTCCCG-3'
hCLDN3	5'-TCGGCCAACACCATTATCCG-3'	5'-CCGTGTACTTCTTCTCGCGT-3'
hCLDN4	5'-GTGATAGTGCCGGTGTCCTG-3'	5'-GCAGCAGAATACTTGGCGGA-3'
hCLDN6	5'-ATGGAACTAATGCGGAGGCT-3'	5'-AGAGACCTGAGTAGGATGGGG-3'
hCLDN7	5'-CCCTAATGGTGGTCTCCCTG-3'	5'-TCTTCACTTTGTCGTCTCCCC-3'
hCLDN8	5'-GCTCTGTTCTGCTGCGTTTT-3'	5'-TTTTTGGGTTGTGCGATGGG-3'
hCLDN9	5'-GGCTTCGACCGGCTTATGA-3'	5'-ACGATGCTGTTGCCGATGA-3'
hCLDN10	5'-GCTCCGATAAAGCCAAAGC-3'	5'-GCTCCTGCCCATCCAATAAAC-3'
hCLDN11	5'-CTGATGATTGCTGCCTCGGT-3'	5'-CGCACACAGGGAACCAGATG-3'
hCLDN12	5'-GGTATGACGGGAGCAGTGAC-3'	5'-CTGAGGGGTAGGGCAAACTG-3'
hCLDN14	5'-GTCCCTCTCGCTCATTGGTG-3'	5'-GGTGTTTGCAGTGGTCGTG-3'
hCLDN15	5'-AGGCTCCAGTGTCCGTGATG-3'	5'-CGTAGGCGTTTCTGCCGTAT-3'
hCLDN16	5'-TGCCTTTTTCTCTGCTGGGT-3'	5'-GCTCCGCAAGTATGGAATCG-3'
hCLDN17	5'-ATTGGAGGGGGTCTGCTTTG-3'	5'-TTCGCTTATCTGTGTGTGGCA-3'
hCLDN18	5'-CATTTGGTGCGGCTCTGTTC-3'	5'-AGTTGGTTTCTTCTGGTGCC-3'
hCLDN19	5'-GTCCTCAGCGTAGTTGGCAT-3'	5'-CAGGTGTGCTTGGGTTGAAG-3'
hCLDN20	5'-ACCTCCACCAAGCTAATGACA-3'	5'-CCTGCTGAGGCCATGATGTTA-3'
hCLDN22	5'-GGTTGGCGGCATTATTTCCC-3'	5'-GCCAAACTAACTCCTGCCCT-3'
hCLDN23	5'-TACAACCACTTCTTGGGGGA-3'	5'-CACTTGGATGGTGCTGACG-3'
hCLDN24	5'-CTGTTTCTGGGCTGGTTTGC-3'	5'-TGAGTTTGCATTTGCGCCAC-3'
hCLDN25	5'-TTCTTGTCTCTGCCCCAGGA-3'	5'-CCGCCTCTTGAATACCCATCT-3'
hCLDN26	5'-TGGGGTCCATCAATCGCA-3'	5'-CGGCAGCAGAATCACAAATG-3'
hCLDN27	5'-GCGACTACTGGTACATCCTGG-3'	5'-TCTCACTGGCAAAAGGGTCG-3'
SLCO1B1	5’-AAACAGCAGAGGCACAACCT-3’	5’-GCTTCCGTCAATAAAACCAACA-3’
SLCO1B3	5’-ATAGCCCTGAAGTGTTGTCCC-3’	5’-TTGTTGCTGATGCTTGGTTTGA-3’
SLC17A1	5’-GGACATGAACCTCAGCATTGTG-3’	5’-GCCGTTTGGATTTGAGGACG-3’
SLC22A7	5’-CTGATACGGGACTGGCGATG-3’	5’-CCTGTGGGCCTCTTTCACAT-3’
SLC22A1	5’-TCCTCTTCCTGCTCTACTACTGG-3’	5’-CCCATTCTTTTGAGCGATGT-3’
ABCC2	5’-ACGGGCACATCACCATCAAG-3’	5’-CCAGGCAGCATTTCCAAGTC-3’
ABCB1	5’-ATGAAGTTGAATTAGAAAATGCAG-3’	5’-GGAAACTGGAGGTATACTTTCATC-3’
ABCB11	5’-AGCGATTCTATGACCCCTGTG-3’	5’-GGCTCTTGCTCCACTATCCC-3’
CYP1A2	5’-GAATGGCTTCTACATCCCCA-3’	5’-TCATCTTCTCACTCAAGGGCT-3’
CYP2B6	5’-ACATCATCCCCAAGGACACAGA-3’	5’-GCATCCAGAAAGTGGTCAGG-3’

CYP2C9	5’-AGGAAAAGCACAACCAACCAT-3’	5’-CAGCAGGAGAAGGAGAGCATA-3’
CYP2C19	5’-GGATTGTAAGCACCCCCTG-3’	5’-TAAAGTCCCGAGGGTTGTTG-3’
CYP2D6	5’-CTCACATGCCCTACACCACT-3’	5’-GTGATGAGTGTCGTTCCCT-3’
CYP2E1	5’-GGAACTATGGGATGGGGAAAC-3’	5’-CGGAAGAGGATGTCGGCTAT-3’
CYP3A4	5’-TATGGAAAAGTGTGGGGCTT-3’	5’-TCCGGTTTGTGAAGACAGAAT-3’
UGT1A1	5’-TCACCAAAATCCACTATCCCA-3’	5’-GTTCGCAAGATTCGATGGTC-3’
CES1	5’-GCTCTTGGAGACGACATTGA-3’	5’-CATCCCATCAATCACAGTGC-3’
ACTB	5’-ACTCTTCCAGCCTTCCTTC-3’	5’-AGCACTGTGTTGGCGTACA-3’

Supplementary Table 5: LC-MS/MS detection conditions for test compounds.

Compound	RT (min)	Ion mode	m/z monitored	CE (V)
Acetaminophen	1.40	Positive	152.0 > 110.0	−9
Cimetidine	1.11	Positive	253.1 > 159.0	−14
Candesartan	2.36	Positive	441.0 > 263.0	−14
Candesartan cilexetil	2.57	Negative	609.1 > 521.1	18
Erythromycin	2.39	Positive	734.4 > 576.3	−19
Hydroxybupropion	1.95	Positive	256.0 > 238.0	−13
1’-hydroxybufuralol	1.89	Positive	278.0 > 186.0	−19
1’-hydroxymidazolam	2.22	Positive	342.0 > 203.0	−26
4’-hydroxydiclofenac	2.88	Positive	312.0 > 230.0	−32
4’-hydroxymephenytoin	2.15	Positive	235.1 > 150.1	−17
6’-hydroxychlorzoxazone	2.05	Negative	184.0 > 119.9	19
Methotrexate	2.13	Positive	455.2 > 308.1	−18
Nafcillin	2.87	Positive	415.2 > 199.1	−13
SN-38	2.53	Positive	393.3 > 349.2	−29
SN-38G	2.32	Positive	569.1 > 393.2	−39
Taurocholate	2.83	Negative	514.1 > 124.0	55
Vincristine	2.28	Positive	825.5 > 765.5	−38
Niflumic acid (IS for metabolic assay)	3.30	Positive	283.3 > 265.2	−21
Propranolol (IS for permeation assay)	2.34	Positive	230.2 > 116.2	−14

IS: Internal standard, RT: Retention time, CE: Collision energy
