# Supplementary material for: Induction of open-form bile canaliculus formation by hepatocytes for evaluation of biliary drug excretion
Source: Commun Biol. 2023 Aug 22;6:866. doi: 10.1038/s42003-023-05216-z (PMC10444810; doi:10.1038/s42003-023-05216-z)
Supplement: Supplementary file 5 — Reporting Summary [file 42003_2023_5216_MOESM5_ESM.pdf]

nature portfolio

Corresponding author(s): Ikumi Tamai

Last updated by author(s): Jul 19, 2023

# Reporting Summary

Nature Portfolio wishes to improve the reproducibility of the work that we publish. This form provides structure for consistency and transparency in reporting. For further information on Nature Portfolio policies, see our Editorial Policies and the Editorial Policy Checklist.

## Statistics

For all statistical analyses, confirm that the following items are present in the figure legend, table legend, main text, or Methods section.

| n/a | Confirmed | |
|---|---|---|
| ☐ | ☒ | The exact sample size ($n$) for each experimental group/condition, given as a discrete number and unit of measurement |
| ☐ | ☒ | A statement on whether measurements were taken from distinct samples or whether the same sample was measured repeatedly |
| ☐ | ☒ | The statistical test(s) used AND whether they are one- or two-sided<br>*Only common tests should be described solely by name; describe more complex techniques in the Methods section.* |
| ☒ | ☐ | A description of all covariates tested |
| ☒ | ☐ | A description of any assumptions or corrections, such as tests of normality and adjustment for multiple comparisons |
| ☐ | ☒ | A full description of the statistical parameters including central tendency (e.g. means) or other basic estimates (e.g. regression coefficient) AND variation (e.g. standard deviation) or associated estimates of uncertainty (e.g. confidence intervals) |
| ☐ | ☒ | For null hypothesis testing, the test statistic (e.g. $F$, $t$, $r$) with confidence intervals, effect sizes, degrees of freedom and $P$ value noted<br>*Give P values as exact values whenever suitable.* |
| ☒ | ☐ | For Bayesian analysis, information on the choice of priors and Markov chain Monte Carlo settings |
| ☒ | ☐ | For hierarchical and complex designs, identification of the appropriate level for tests and full reporting of outcomes |
| ☐ | ☒ | Estimates of effect sizes (e.g. Cohen's $d$, Pearson's $r$), indicating how they were calculated |

*Our web collection on statistics for biologists contains articles on many of the points above.*

## Software and code

Policy information about availability of computer code

| | |
|---|---|
| Data collection | LC/MS: LabSolutions (Shimadzu) and Microscopy: LSM710 (Carl Zeiss) |
| Data analysis | LC/MS: LabSolutions (Shimadzu), Microscopy: LSM710 (Carl Zeiss) or ImageJ and Excel (Microsoft) |

For manuscripts utilizing custom algorithms or software that are central to the research but not yet described in published literature, software must be made available to editors and reviewers. We strongly encourage code deposition in a community repository (e.g. GitHub). See the Nature Portfolio guidelines for submitting code & software for further information.

## Data

Policy information about availability of data

All manuscripts must include a data availability statement. This statement should provide the following information, where applicable:

- Accession codes, unique identifiers, or web links for publicly available datasets
- A description of any restrictions on data availability
- For clinical datasets or third party data, please ensure that the statement adheres to our policy

Antibody list for immunofluorescence and western blot analysis are available in Supplementary Table 3. Primers used for quantitative PCR are available in Supplementary Table 4. LC/MS data and the analyzed m/z values are available in Supplementary Table 5.

## Human research participants

Policy information about studies involving human research participants and Sex and Gender in Research.

| | |
|---|---|
| Reporting on sex and gender | N/A |
| Population characteristics | N/A |
| Recruitment | N/A |
| Ethics oversight | N/A |

Note that full information on the approval of the study protocol must also be provided in the manuscript.

# Field-specific reporting

Please select the one below that is the best fit for your research. If you are not sure, read the appropriate sections before making your selection.

☒ Life sciences ☐ Behavioural & social sciences ☐ Ecological, evolutionary & environmental sciences

For a reference copy of the document with all sections, see nature.com/documents/nr-reporting-summary-flat.pdf

# Life sciences study design

All studies must disclose on these points even when the disclosure is negative.

| | |
|---|---|
| Sample size | We used three biological replicate samples for acquisition of error bars indicating standard deviation. |
| Data exclusions | We did not exclude any data from analysis. |
| Replication | We performed two independent experiments and confirmed that the findings were reproducible. |
| Randomization | Since comparisons between biological samples were not performed, randomization is not relevant to our study. |
| Blinding | Since comparisons between biological samples were not performed, binding is not relevant to our study. |

# Reporting for specific materials, systems and methods

We require information from authors about some types of materials, experimental systems and methods used in many studies. Here, indicate whether each material, system or method listed is relevant to your study. If you are not sure if a list item applies to your research, read the appropriate section before selecting a response.

### Materials & experimental systems

| n/a | Involved in the study | |
|---|---|---|
| ☐ | ☒ | Antibodies |
| ☐ | ☒ | Eukaryotic cell lines |
| ☒ | ☐ | Palaeontology and archaeology |
| ☒ | ☐ | Animals and other organisms |
| ☒ | ☐ | Clinical data |
| ☒ | ☐ | Dual use research of concern |

### Methods

| n/a | Involved in the study | |
|---|---|---|
| ☒ | ☐ | ChIP-seq |
| ☒ | ☐ | Flow cytometry |
| ☒ | ☐ | MRI-based neuroimaging |

## Antibodies

Antibodies used

- Anti-MRP2 antibody, mouse IgG monoclonal (GTX23373), Gene Tex.
- Anti-claudin-1 antibody, mouse IgG monoclonal (sc-81796), Santa Cruz Biotechnology.
- Anti-claudin-2 antibody, rabbit IgG polyclonal (ab53032), abcam.
- Anti-claudin-3 antibody, rabbit IgG monoclonal (ab214487), abcam.
- Anti-claudin-9 antibody, mouse IgG monoclonal (sc-398836), Santa Cruz Biotechnology.
- Anti-P-gp antibody, rabbit IgG monoclonal (13342S), Cell signaling Technology.

- Anti-BSEP antibody, rabbit IgG monoclonal (HPA019035), Sigma-Aldrich.
- Anti-OATP1B1 antibody, mouse IgM monoclonal (ab15441), abcam.
- Anti-OATP1B3 antibody, rabbit IgG polyclonal (HPA004943), Sigma-Aldrich.
- Anti-NTCP antibody, rabbit IgG polyclonal (ab131084), abcam.
- Anti-OCT1 antibody, mouse IgG monoclonal (NBP1-51684), Novus Biologicals.
- Goat anti-mouse Alexa Fluor 594 (A11005), Thermo Fisher Scientific.
- Goat anti-mouse IgG (H+L) secondary antibody HRP (62-6520), Thermo Fisher Scientific.
- Goat anti-rabbit IgG (H+L) secondary antibody HRP (65-6120), Thermo Fisher Scientific.
- Donkey anti-rabbit Alexa Fluor 488 (A21206), Thermo Fisher Scientific.
- Goat anti-mouse Alexa Fluor 488 (A11001), Thermo Fisher Scientific.
- Goat anti-mouse Alexa Fluor 488 (ab150121), abcam.

Validation

- Anti-MRP2 antibody (GTX23373): https://www.genetex.com/Product/Detail/MRP2-antibody-M2III-6/GTX23373
- Anti-claudin-1 antibody (sc-81796): https://www.scbt.com/ja/p/claudin-1-antibody-xx7
- Anti-claudin-2 antibody (ab53032): https://www.abcam.co.jp/claudin-2-antibody-ab53032.html
- Anti-claudin-3 antibody (ab214487): https://www.abcam.co.jp/claudin-3-antibody-epr19971-ab214487.html
- Anti-claudin-9 antibody (sc-398836): https://www.scbt.com/ja/p/claudin-9-antibody-e-7
- Anti-P-gp antibody (13342S): https://en.cellsignal.jp/products/primary-antibodies/mdr1-abcb1-e1y7b-rabbit-mab/13342?%2525252525253BNtt=Pairs&N=0+4294956287&Nrpp=60&No=0&fromPage=plp
- Anti-BSEP antibody (HPA019035): https://www.sigmaaldrich.com/JP/ja/product/sigma/hpa019035
- Anti-OATP1B1 antibody (ab15441): https://www.abcam.co.jp/oatp1b1-antibody-esl-ab15441.html
- Anti-OATP1B3 antibody (HPA004943): https://www.sigmaaldrich.com/JP/ja/product/sigma/hpa004943
- Anti-NTCP antibody (ab131084): https://www.abcam.co.jp/slc10a1ntcp1-antibody-ab131084.html
- Anti-OCT1 antibody (NBP1-51684): https://www.novusbio.com/products/slc22a1-antibody-2c5_nbp1-51684

# Eukaryotic cell lines

Policy information about cell lines and Sex and Gender in Research

| | |
|---|---|
| Cell line source(s) | All cell line were obtained from ATCC. |
| Authentication | None of the cell lines have been authenticated in our lab. |
| Mycoplasma contamination | All cell lines tested negative for Mycoplasma contamination. |
| Commonly misidentified lines (See ICLAC register) | None |
